# Novel reference genes for quantifying transcriptional responses of *Escherichia coli *to protein overexpression by quantitative PCR

**DOI:** 10.1186/1471-2199-12-18

**Published:** 2011-04-23

**Authors:** Kang Zhou, Lihan Zhou, Qing 'En Lim, Ruiyang Zou, Gregory Stephanopoulos, Heng-Phon Too

**Affiliations:** 1 Chemical and Pharmaceutical Engineering, Singapore-MIT Alliance, 4 Engineering Drive 3, Singapore; 2 Department of Biochemistry, National University of Singapore, 8 Medical Drive, Singapore; 3 Department of Chemical Engineering, Massachusetts Institute of Technology, 77 Massachusetts Avenue, Cambridge, The USA

## Abstract

**Background:**

Accurate interpretation of quantitative PCR (qPCR) data requires normalization using constitutively expressed reference genes. Ribosomal RNA is often used as a reference gene for transcriptional studies in *E. coli*. However, the choice of reliable reference genes has not been systematically validated. The objective of this study is to identify a set of reliable reference genes for transcription analysis in recombinant protein over-expression studies in *E. coli*.

**Results:**

In this study, the meta-analysis of 240 sets of single-channel Affymetrix microarray data representing over-expressions of 63 distinct recombinant proteins in various *E. coli *strains identified twenty candidate reference genes that were stably expressed across all conditions. The expression of these twenty genes and two commonly used reference genes, *rrsA *encoding ribosomal RNA 16S and ihfB, was quantified by qPCR in *E. coli *cells over-expressing four genes of the 1-Deoxy-D-Xylulose 5-Phosphate pathway. From these results, two independent statistical algorithms identified three novel reference genes *cysG*, *hcaT*, and *idnT *but not *rrsA *and *ihfB *as highly invariant in two *E. coli *strains, across different growth temperatures and induction conditions. Transcriptomic data normalized by the geometric average of these three genes demonstrated that genes of the lycopene synthetic pathway maintained steady expression upon enzyme overexpression. In contrast, the use of rrsA or ihfB as reference genes led to the mis-interpretation that lycopene pathway genes were regulated during enzyme over-expression.

**Conclusion:**

This study identified *cysG/hcaT/idnT *to be reliable novel reference genes for transcription analysis in recombinant protein producing *E. coli*.

## Background

Recently, transcriptomic studies using DNA microarray and qPCR identified gene expression changes in *E. coli *[1-3]. Accurate quantification of transcriptomic changes requires reliable normalization methods to minimize technical variations, such as the quality/quantity of samples and instrumental bias. To date, normalization with internal reference genes is the most frequently used and reliable method for qPCR data [4,5]. To the best of our knowledge, there has been no systematic study to identify reference genes for qPCR in *E. coli*. To date, *rrsA *encoding ribosomal RNA 16S [6,7] and *ihfB *[2,8-13] are the two most frequently used reference genes in *E. coli*. However, the stability of these two genes has not been validated.

*E. coli *has been extensively used in biotechnology for the production of proteins, therapeutic metabolites, and biofuels [1,14,15]. Recombinant DNA technology has provided various means to express proteins with diverse functions and for the over-production of metabolites in *E. coli*. As a result, a set of invariant reference genes for qPCR normalization during recombinant protein production is highly desired in *E. coli*.

In the present study, we aim to identify and validate a set of reference genes for the accurate normalization of transcription analysis in recombinant protein producing *E. coli *cells. Candidate reference genes were systematically selected from public microarray database. The temporal expressions of these twenty genes, *rrsA *and *ihfB *were quantified in two different *E. coli *strains induced to express enzymes of the 1-deoxy-D-xylulose 5-phosphate (DXP) pathway at two different temperatures. Two independent statistical algorithms 'geNorm' [4] and 'NormFinder' [5] were utilized to identify reliable reference genes stably expressed under the conditions tested. Further analysis examined if normalization factors derived from these novel reference genes or that of *rrsA *or *ihfB *allowed accurate quantification of the expressions of genes producing lycopene. This study illustrates the importance of the use of validated reference genes in transcriptional studies in *E. coli*.

## Results

### High protein overexpression inhibits metabolite production

**Figure 1:**
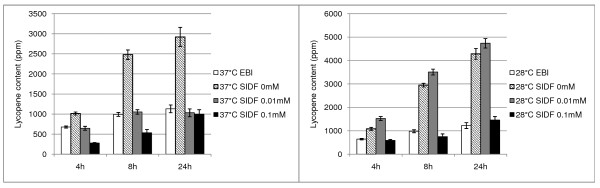
**Time course of the production of lycopene (ppm) in *E. Coli *BL21 harboring p20T7MEP on various conditions**. 37°C/28°C EBI indicates the cells harboring pAC-LYC only without induction at 37°C or 28°C; 37°C/28°C SIDF 0 mM indicates the cells harboring p20T7MEP and pAC-LYC without induction at 37°C or 28°C; 37°C/28°C SIDF 0.01 mM indicates the cells harboring p20T7MEP and pAC-LYC with 0.01 mM IPTG induction at 37°C or 28°C; 37°C/28°C SIDF 0.1 mM indicates the cells harboring p20T7MEP and pAC-LYC with 0.1 mM IPTG induction at 37°C or 28°C; The standard errors were calculated based on four biological replicates.

BL21 (DE3), a widely used *E. coli *strain for recombinant protein production [16], has been used to produce lycopene, an natural antioxidant [17]. The lycopene precursors, isopentenyl diphosphate (IPP) and dimethylallyl diphosphate (DMAPP), are produced via the DXP pathway which can be increased by the expressions of four rate limiting enzymes, *dxs*, *idi*, *ispD*, and *ispF *[18]. To increase lycopene production, these four enzymes were expressed in BL21 cells at 28°C and 37°C (Figure 1, and additional file 1: supplementary figure S1). The expression of these enzymes under non-induced condition (without IPTG induction) was found to increase lycopene production at 37°C. However, when expression was induced with IPTG induction (0.01 or 0.1 mM), lycopene production was significantly lower as compared to non-induced conditions. At a lower temperature (28°C), non-induced and mild IPTG induction (0.01 mM) but not strong IPTG induction (0.1 mM) enhanced lycopene production. This finding is consistent with previous observations of the inhibitory effects of high induction on lycopene production [17,19,20]. In order to test the hypothesis that this inverse correlation of lycopene production with increasing induction may simply be due to the decrease in the expressions of genes involved in the lycopene pathway, we measured the transcriptional changes by qPCR. In the process of carrying out such measurements, we were confronted with the critical issue of selecting reliable reference genes for normalization of transcriptional changes.

### Selection of candidate reference genes from microarray

A suitable reference gene should be stably expressed across all experimental conditions used in the study. Such reference genes are commonly identified by analyzing the expression stabilities of a pool of pre-selected candidate genes. However, the selection of such candidates poses a major challenge without *a priori *knowledge or assumptions of their stabilities. To identify suitable candidate genes, we surveyed five major microarray databases (Many Microbe Database M^3D^, NIH-GEO, Stanford Microarray Database, ExpressDB and Array Express) for projects pertinent to protein over-expression studies in *E. coli*. To minimize platform specific biases and variations caused by different data normalization strategy, only the uniformly normalized Affymetrix microarray data from M^3D ^database (E_coli_v4_Build_6) were selected [21]. The 240 sets of microarray data from M^3D ^database comprise over-expression studies of a total of 63 recombinant proteins classified into four major functional categories (Additional file 2), providing a broad spectrum of experimental conditions in which stably expressed reference genes can be identified. Among the 4297 genes expressed across all arrays, the top fifty genes were selected based on their low coefficient of variation of signal intensities. These fifty genes were then further analyzed for their genetic loci and functional classifications [22]. Genes that belong to the same transcription unit were excluded and genes from diverse functional classes were selected to avoid potential transcriptional co-regulation. Twenty genes were short-listed as candidate reference genes for further analyses (Table 1).

**Table 1:** Selection of candidate reference genes from microarray data.

Gene symbol	Definition	Average intensity	Primary multi-fun term
ssrA	tmRNA	13.88	cell processes -> protection -> drug resistance/sensitivity
rnpB	RnpB RNA; catalytic subunit of RNAse P	13.42	information transfer -> RNA related -> RNA degradation
pflC	probable pyruvate formate lyase 2 activating enzyme	8.650	information transfer -> protein related -> posttranslational modification
hycG	hydrogenase 3 and formate hydrogenlyase complex	8.09	metabolism -> energy metabolism, carbon -> anaerobic respiration
uxuB	D-mannonate oxidoreductase	8.94	metabolism -> carbon utilization -> carbon compounds
ygjD	YgjD, target for YeaZ protease	9.74	regulation -> type of regulation -> posttranscriptional -> proteases, cleavage of compounds
uxuR	UxuR-fructuronate	8.53	information transfer -> RNA related -> Transcription related
yajR	YajR MFS transporter	8.54	cell processes -> protection -> drug resistance/sensitivity
asnA	asparagine synthetase A	8.78	metabolism -> biosynthesis of building blocks -> amino acids -> asparagine
hcaT	HcaT MFS transporter	8.292	cell structure -> membrane
ldnT	L-idonate/5-ketogluconate/gluconate transporter	8.991	cell structure -> membrane
yghB	conserved inner membrane protein	9.01	cell structure -> membrane
ugpQ	glycerophosphodiester phosphodiesterase, cytosolic	9.16	metabolism -> central intermediary metabolism -> misc. glycerol metabolism
metL	aspartate kinase/homoserine dehydrogenase	8.973	metabolism -> biosynthesis of building blocks -> amino acids -> homoserine
pbpC	putative peptidoglycan enzyme	8.73	cell structure -> murein
ilvY	IlvY DNA binding transcriptional dual regulator	9.320	information transfer -> RNA related -> Transcription related
phnN	ribose 1,5-bisphosphokinase	8.05	metabolism -> metabolism of other compounds -> phosphorous metabolism
cysG	uroporphyrin III C-methyltransferase [multifunctional]	8.219	metabolism -> biosynthesis of building blocks -> cofactors, small molecule carriers -> heme, porphyrine
cca	tRNA nucleotidyltransferase	9.19	information transfer -> RNA related -> RNA modification
**rrsA**	**rrsA 16S ribosomal RNA**	**14.6**	**cell structure -> ribosomes**

**ihfB**	**integration host factor β subunit**		

Meta-analysis of the expression profiles of 240 arrays relevant to recombinant protein production. Twenty candidate reference genes were selected based on the lowest CV. The log2 transformed values of the average signal intensities among the 240 arrays were shown as Mean. ihfB was included for comparison, and commonly used reference genes were highlighted.

### Temporal expression of candidate reference genes in *E. coli *cells over-expressing metabolic pathway genes

**Figure 2:**
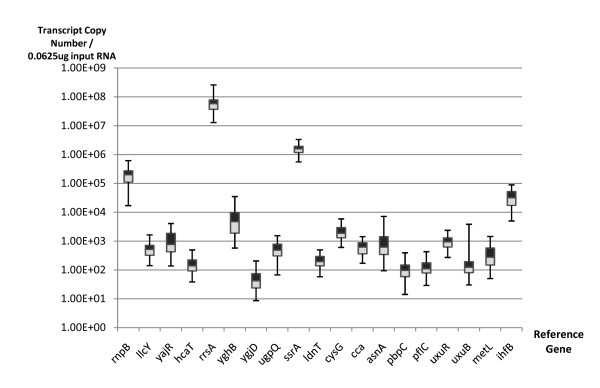
**Distribution of the expression levels of genes examined in *E. Coli *BL21 (DE3)**. Box plot representation of the expression levels of seventeen candidate reference genes and two commonly used house-keeping genes among the 72 biological samples. The expression level of each gene was represented as the absolute copy number per unit input total RNA (0.0625 μg), quantified by qPCR using serial dilutions of standards. Primer design, assay efficiency and intra- and inter-assay variations were reported in supplementary data (Additional file 1: supplementary table 1).

The temporal expressions (0 h, 4 h & 8 h after IPTG induction) of the 20 candidate reference genes, 2 commonly used reference genes and 7 lycopene synthetic genes were analyzed by qPCR (*rrsA *encoding ribosomal RNA 16S is both a commonly used reference gene and selected gene from the meta-analysis of microarray data). All but two of the candidate reference genes were expressed at detectable levels in BL21 cells. The absolute levels of expressions of each gene were interpolated from standard curves and the expression levels of these genes spanned over 6 logs (Figure 2 and additional file 1: supplementary figure S2). All the qPCR assays showed high efficiency of amplification (>90%) and low intra- and inter- assay variations (Additional file 1: supplementary table S1).

### Stabilities of candidate reference genes and common housekeeping genes

Two independent statistical algorithms, geNorm and NormFinder, were used to analyze the gene expressions. geNorm is based on pairwise variation analysis and assumes none of the reference genes are co-regulated. NormFinder is a model based method that analyzes the stability of each candidate gene, assuming all input samples are of equal quality and quantity. The "M value" (geNorm) or "Stability Value" (NormFinder) are inversely correlated to the stability of the candidate gene. The stability of the 17 candidate genes, *rrsA *and *ihfB *across all experimental conditions was analyzed by the two methods. Interestingly, both approaches identified *cysG*, *idnT*, and *hcaT *as the most stable reference genes (Additional file 1: supplementary figure S3). On the contrary, *rrsA *and *ihfB *were poorly ranked (Additional file 1: supplementary figure S3). In addition, pairwise variation analysis by geNorm showed that the combination of *cysG*, *idnT*, and *hcaT *is sufficiently stable (V3/4 = 0.116, less than the proposed cut-off of 0.15) to serve as normalizer.

Further analysis of candidate gene stabilities in cells grown at different temperatures or at specific time point (Additional file 1: supplementary table S2) revealed that the stability rankings of candidate genes do vary among different subgroups. However, *cysG*, *idnT *and *hcaT *were consistently more stable than *rrsA *and *ihfB *under all subgroups examined. The results suggested that the three novel candidate genes may serve as better normalizers than the commonly used *rrsA *and *ihfB *for gene expression profiling in BL21 cells.

### Comparison of the normalization factors generated by different reference gene(s)

**Figure 3:**
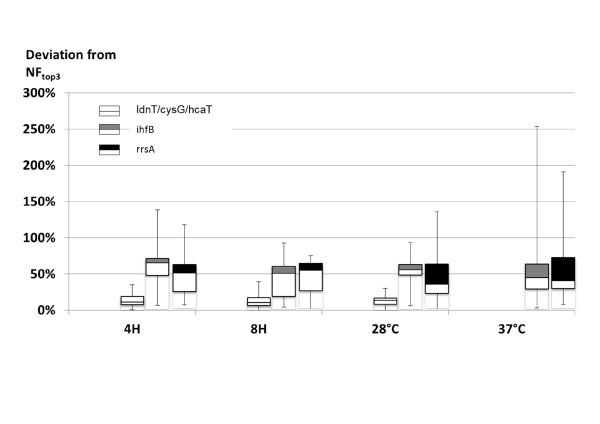
**Comparison of the normalization factors calculated using different reference gene(s) in *E. Coli *BL21 (DE3)**. Normalization factors (NFs) calculated with idnT/cysG/hcaT, rrsA and ihfB were compared to that calculated by the top 3 reference genes (NF_top3_) as recommended by both NormFinder and geNorm, for each stimulus. The percentage deviations of NF_ldnT/cysG/hcaT_; NF_rrsA_; NF_ihfB _from NF_top3 _(|NF_x_-NF_top3_|/NF_top3_) were represented by box plot. The 25^th ^percentile to the 75^th ^percentile (boxes), and ranges (whiskers) were shown.

To account for sample to sample variations introduced during RNA isolation and quantification, raw expression profiles of target genes were scaled by a normalization factor (NF) calculated based on independent measurement of one or more internal reference genes. When multiple genes are used, NF is the geometric average of the relative expression level of each gene [4]. (An excel template has been included as additional file 3 for calculating the geometric average) As we have previously shown [23], NF based on an unstable reference gene could differ significantly from the NF of a stable gene, which resulted in misinterpretation of normalized target gene expression. To test the robustness of the three novel genes in each subgroup, the NFs computed based on *cysG*, *idnT *and *hcaT *(NF_cysG/idnT/hcaT_) were compared to the NFs based on the three most stable genes in each subgroup (NF_top3_), e.g. *cysG*, *ssrA *and *pflC *(subgroup 8H). Similarly, the deviations of NF_rrsA _and NF_ihfB _from NF_top3 _were examined. Remarkably, NF_cysG/idnT/hcaT _differed from NF_top3 _by less than 40% in all subgroups examined, whereas NF_rrsA _deviated from NF_top3 _by as much as 290% (Figure 3). Thus, the expression stabilities of *cysG*, *idnT*, and *hcaT *were sufficient to warrant their use as reference genes in each experiment subgroup. On the contrary, the analysis further highlighted the instability of *rrsA *and *ihfB *and suggested that the use of *rrsA *and *ihfB *as reference genes were likely to lead to significant misinterpretation of lycopene synthetic gene profile.

### Choice of reference genes affects the interpretation of target gene regulation

**Figure 4:**
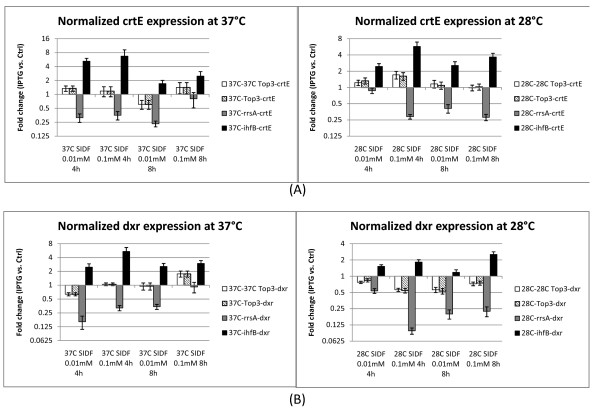
**Fold changes in target gene expressions normalized using different reference gene(s) in *E. Coli *BL21 (DE3)**. Fold changes in transcript expressions of crtE (A) and dxr (B) in IPTG induced cells relative to that of control were normalized by (1) geometric mean of top three most stable genes in the temperature subgroup; (2) geometric mean of idnT, cysG, and hcaT; (3) rrsA or (4) ihfB. Normalization by rrsA falsely identified the downregulation of all target genes, and normalization by ihfB falsely identified the upregulation of all target genes. 2 is the threshold for upregulation and 0.5 is the threshold for downregulation.

To demonstrate that the use of unstable reference genes could substantially alter the interpretation of target gene expression profiles, we examined the target gene profiles normalized by four NFs: NF_top3 _(Normalization factors derived from top3 genes in each subgroup), NF_cysG/idnT/hcaT_, NF_rrsA _and NF_ihfB_. (Figure 4 and additional file 1: supplementary figure S4). No significant difference was observed in target gene expressions normalized by NF_cysG/idnT/hcaT _or NF_top3_. In both cases, lycopene synthetic genes were found to be stably expressed upon IPTG induction. The results showed the reduction of lycopene production at high induction was not due to limited transcription of lycopene synthetic genes. In comparison, different conclusions were derived when the results were normalized with *rrsA *or *ihfB*. Lycopene synthetic genes appeared to be significantly downregulated or upregulated when *rrsA *or *ihfB *were used as normalizers, respectively. Such misinterpretation was caused by the up-regulation of *rrsA *and the down-regulation of *ihfB *expressions upon IPTG induction (Additional file 1: supplementary figure S5). A detailed time course analysis of *rrsA *and *ihfB *expression further confirmed that they were regulated during recombinant enzyme production. At 0.5 h after IPTG induction, *rrsA *and *ihfB *were not regulated but the expressions were significantly altered at 2 h and 4 h after IPTG induction (Additional file 1: supplementary figure S6).

### Further validation of novel reference genes in an *E. coli *K-12 derivative strain

Besides *E. coli *BL21 (DE3), *E. coli *K-12 derivatives were also frequently used for recombinant protein production and metabolic engineering. Unlike BL21 (DE3) utilizing T7 system from bacteriophage, the K-12 derivatives employ endogenous expression system. To test if the same set of reference genes is applicable across strains, the expression stabilities of the 15 candidate genes (two genes were not detectable in the newly tested strain as compared to BL21), *rrsA *and *ihfB *were evaluated in a K-12 derivative, DH10B. Similar to that of BL21, DH10B cells were engineered to over-express dxs, idi, ispD, and ispF controlled by araBAD system at 28°C. Sample preparation and data analysis were performed as described for BL21.

**Figure 5:**
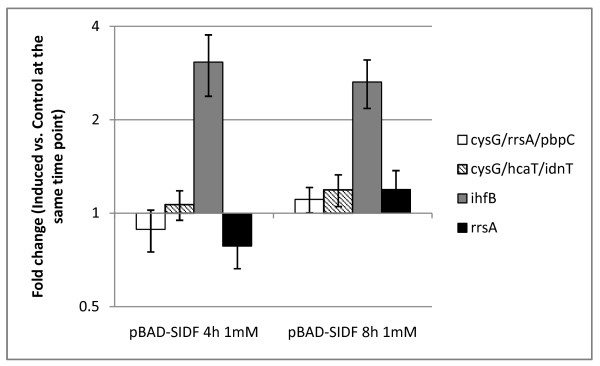
**Fold changes in crtE expressions normalized using different reference gene(s) in *E. coli *DH10B**. Fold changes in transcript expressions of crtE in IPTG induced cells relative to that of control were normalized by (1) geometric mean of top three most stable genes in *E. Coli *DH10B - cysG/rrsA/pbpC; (2) geometric mean of top three most stable genes in *E. Coli *BL21 - idnT, cysG, and hcaT; (3) rrsA or (4) ihfB. 2 is the threshold for upregulation and 0.5 is the threshold for downregulation.

When analyzed by Normfinder and geNorm, the three most stable groups of genes in DH10B were *cysG*/*rrsA*/*pbpC *and *cysG*/*rrsA*/*IIcY*, respectively (Additional file 1: supplementary table S3). To test if the use of *cysG*/*idnT*/*hcaT *as normalizers was still applicable, NF_cysG/idnT/hcaT _was compared with NF_cysG/rrsA/pbpC _in DH10B. No statistically significant difference was found (data not shown). The transcript levels of target gene *crtE *normalized by the two sets of reference genes were also comparable (Figure 5). These suggested that the combination of *cysG*/*hcaT*/*idnT *is indeed stable in DH10B cells to provide accurate interpretation of qPCR results. The results showed that lycopene synthetic genes were not transcriptionally regulated (less than 2 fold change) after induction in DH10B. Consistent with results in BL21, normalization with an unstable reference gene *ihfB *overestimated the upregulation of *crtE *transcription (Figure 5). Finally, it is worthy to note that unlike in BL21, *rrsA *was stably expressed in DH10B (Figure 5 and additional file 1: supplementary table S3), suggesting that the expression systems and genetic differences among *E. coli *strains can affect the choices of qPCR reference genes.

## Discussion

Normalization of gene expression is critical for the accurate interpretation of transcriptional changes determined by qPCR. The use of total RNA as a reference has been explored as an alternative method to normalize qPCR gene expression but this approach was found not to be suitable as ribosomal RNA, the main constituent, was found to be regulated [24-26]. The use of multiple reference genes is deemed as the preferred method, where technical variations are taken into account [4,5]. In this study we aimed to identify reliable reference genes for qPCR in recombinant protein producing *E. coli*, since over expression of recombinant proteins in *E. coli *is extensively used in biotechnology.

We first analyzed single platform and uniformly normalized microarray expression data from a substantial number of recombinant protein over-expression studies. From these, we selected 20 candidate reference genes based on their variation of signal intensities across all selected arrays. The expression of the 20 candidate genes and 2 commonly used reference genes were then measured by qPCR under a variety of conditions, including different strains, growth stages, temperatures, and induction levels. The expression of *cysG*, *idnT *and *hcaT *were found to be most constant as ranked by geNorm and NormFinder. Furthermore, scaling with the geometric average of these three genes (*cysG*/*idnT*/*hcaT*) provided accurate data interpretation under all tested conditions. The genes, *idnT *and *hcaT*, encode transporters for idonate and 3-phenylpropionate, respectively and *cysG *encodes a metabolic enzyme involved in siro-heme synthesis. Putative regulations of these gene expressions were predicted in EcoCyc http://ecocyc.org/, but only the control of *idnT *expression by its substrate idonate was experimentally verified in *E. coli *[27]. Idonate is not a commonly used ingredient in *E. coli *media, which might explain the constant expression of *idnT *in the diverse microarray studies which we have analyzed and the qPCR study carried out herein.

Normalization of gene expression using the scaling factor derived from the three most stable genes (*cysG/hcaT/idnT*) revealed that the induction of the 4 enzymes (*dxs, idi*, *ispD*, and *ispF*) of the DXP pathway did not inhibit the expression of the enzymes involved in the lycopene production. The lycopene biosynthetic genes expressed from multiple copy plasmid and some of endogenous DXP pathway genes were found to maintain constant transcription levels during high induction levels. In contrast to the empirically validated reference genes described here, *rrsA *[6,7] encoding ribosomal RNA 16S and ihfB [2,8-13], were unvalidated but yet commonly used as reference genes in *E. coli*. Disturbingly, we found that *ihfB *and *rrsA *were less stable than *cysG/idnT/hcaT *under many conditions tested. The expression of lycopene biosynthetic genes normalized with *ihfB *appeared to be upregulated by up to 5 fold during induction, while *rrsA *overestimated gene downregulation by up to 8 fold (Figure 4 and additional file 1: supplementary figure S4). This type of incorrect interpretation can lead to faulty conclusions with regard to transcriptional regulations and is especially problematic in guiding further genetic manipulations. These results showed that the *E. coli *transcription machinery is fairly robust, and the lycopene production inhibition is not due to altered transcriptions of genes in the pathway examined. The results also illustrate the importance of the use of reliable, validated reference genes for qPCR analysis in *E. coli*.

In addition, the stabilities of gene expressions of *rrsA *in two tested strains were not consistent. We found that *rrsA *expression was fairly stable in DH10B but not in BL21 (DE3). This could be due to the strain-specific expression systems of the two cells. BL21 (DE3) was engineered to produce recombinant T7 RNA polymerase for target protein expression whereas DH10B uses *E. coli *endogenous expression systems. Another possibility could be due to the differences in their genetic backgrounds. Nonetheless, this example clearly highlights the necessity of evaluating the suitability of reference genes in different experimental contexts. Extending the study herein, it will be of interest to examine the use of the normalization factor of these three genes (*cysG/hcaT/idnT*) in more specific applications including conditions in biotechnology such as heat/cold-shock stresses, the presence of alcohols, and knockout of metabolic genes, and the study of pathogenic strains.

## Conclusion

This systematic study included the meta-analysis of public microarray data and extensive in house qPCR analysis. Twenty candidate genes were identified and their expression stabilities were analyzed and compared with two commonly used reference genes *rrsA *and *ihfB*. The expression of *cysG/hcaT/idnT *was found to be most constant in the two recombinant protein producing *E. coli *strains across different growth stages, growth temperature, and inducer concentrations. Only the normalization factors derived from *cysG/hcaT/idnT *but not the ones derived from *rrsA *or *ihfB *provided accurate interpretation of transcriptional responses in the tested conditions. The identified reference genes in this study could be useful for other studies involving recombinant protein producing *E. coli*.

## Methods

### Bacteria strains and plasmids

*E. coli *BL21-Gold (DE3) (Stratagen) [*E. coli *B F^- ^ompT hsdS (r_B_^- ^m_B_^-^) dcm^+ ^Tet^r ^gal λ(DE3) endA Hte], and DH10B (New England Biolabs) [araD139 Δ(ara, leu)7697 fhuA lacX74 galK (ϕ80 Δ(lacZ) M15) mcrA galU recA1 endA1 nupG rpsL Δ(mrr-hsdRMS-mcrBC)] were used for lycopene production with pACLYC plasmid [28]. The dxs-idi-ispDF operon was amplified by polymerase chain reaction (PCR) from p20T7MEP [15]. Primers used were SacI-Ec_dxs: GCTTAGAGCTCAGTTTTGATATTGCCAAATA and Ec_ispF-XhoI: GTAACCTCGAGTCATTTTGTTGCCTTAATGA. The purified PCR product was ligated into a modified pBAD-B vector (Invitrogen), engineered to contain SacI & XhoI restriction sites. The resulting plasmid was termed pBAD-SIDF. p20T7MEP was transformed into BL21-Gold (DE3) and pBAD-SIDF was transformed into DH10B.

### Bacteria growth and induction of protein expression

Four colonies were picked from agar plates, inoculated into 2xPY medium (20 g/L Peptone, 10 g/L Yeast extract, and 10 g/L NaCl, pH = 7) containing 34 μg/mL Chloramphenicol and 100 μg/mL Ampicillin, and incubated overnight. Ten micro-liter aliquots of overnight grown cell culture were inoculated into 1 mL 2xPY medium in 14 mL BD Falcon™ tube. Cells were grown at 37°C with shaking (300rpm) till OD595 reached the range of 0.5~1.0. The cells were then induced with various concentrations of L-Arabinose/IPTG and grown at 28°C or 37°C for indicated time before collected for RNA/protein extraction or Lycopene assay.

### Protein assay

Cell suspension, equivalent to 0.5 mL OD595 = 1.0 cells, were withdrawn from each biological replicate 4 h after induction for protein assay. The cells were centrifuged at 20,000 g for 1 min, and pellets were resuspended in 100 μL 2% SDS solution and incubated for 10 min at 95°C, The protein samples were separated on a 12% denaturing polyacrylamide gel and the protein bands were visualized by Instant Blue (Gentaur).

### Lycopene quantification

Twenty microliters of cell suspension were sampled from each biological replicate at 4 h, 8 h, and 24 h after induction, and OD595 was recorded. The cells were centrifuged at 2,800 g for 2 mins, washed twice with PBS, and resuspended in 200 μL acetone. Resuspended cells were vortexed for 10 mins and centrifuged at 2,800 g for 2 mins. One hundred microliters of supernatant was mixed with equal volume of Ethanol and transferred to 96 Well Optical Bottom Plates (NUNC). Lycopene content was determined by interpolating from a standard dilution of lycopene (Sigma), based on absorbance at 472 nm (Spectra Max 190, Molecular Devices). Cell dry weight was estimated from cell density (OD595).

### Meta-analysis of microarray data

Two hundred and forty sets of Affymetrix microarray expression data (RMA normalized) were extracted from the Many Microbe Microarray Databases (M^3D ^E_coli_v4_Build_6). The data belongs to five different recombinant protein over-expression projects (upregulation_low_norfloxacin, upregulation_high_norfloxacin, metabolic_burden_response, recombinant_fermenter, and stringent_response) that include over-expression of 63 different proteins. The mean expression value and standard deviation were computed for each gene across 240 sets of microarray data. The coefficient of variation (CV = Standard Deviation/Mean) of signal intensity was calculated. The top fifty genes with the lowest CV (CV, 0.78% - 2.72%) were selected for further analysis of their genetic loci ecocyc.org and functional classifications [22].

### Primer design

The gene symbol and sequence for each candidate reference gene was retrieved from the affymetrix microarray probe set and compared to the NCBI *E. coli *BL21 (DE3) genomic sequence (NC_012971.1). Vector NTI Advance 10 (Invitrogen) was used to design two sets of primers for each target gene. The first set of primers generated amplicons of ~300 bp which were used as standard templates for qPCR of the targeted gene. The second set of primers was used for qPCR assays and was designed to amplify a ~100 bp region within each ~300 bp template. All products generated after amplifications were verified by gel-electrophoresis.

### RNA purification and cDNA synthesis

Total RNA from *E. coli *was prepared using TRIzol^® ^reagent (Invitrogen) according to the manufacturer's instructions. Total RNA was collected from samples in quadruplicate at each treatment time point. RNA concentration was quantified using a NanoDrop ND-1000 spectrophotometer (Thermo Scientific), and the 260/280 and 260/230 ratios were examined for protein and solvent contamination. The integrities of all RNA samples were confirmed by formaldehyde agarose gel. Eight hundred nanograms of total RNA were reverse transcribed in a total volume of 40 μL containing ImpromII (Promega) for 60 min at 42°C according to the manufacturer's instructions. The reaction was terminated by heating at 70°C for 5 min.

### Quantitative real-time PCR

The cDNA levels were then analyzed using a Biorad iCycler 4 Real-Time PCR Detection System (Bio-Rad) with SYBR Green I detection. Each sample was measured in duplicate in a 96-well plate (Bio-rad) in a reaction mixture (30 μL final volume) containing 1× XtensaMix-SG (BioWORKS), 200 nM primer mix, 2.5 mM MgCl_2_, 0.75 U of iTaq DNA polymerase (iDNA). Realtime PCR was performed with an initial denaturation of 3 min at 95°C, followed by 40 cycles of 20 s at 95°C, 20 s at 60°C, and 20s at 72°C. Fluorescent detection was performed at the annealing phase and during subsequent dissociation curve analysis to confirm that a single product had been amplified. The threshold cycles (Ct) were calculated using the iQ5 Optical system software version 2.0. Primer dimers in all the assays showed distinct melt characteristics from the desired amplicons. All real-time PCR quantifications were performed simultaneously with PCR amplified standards and no-template controls. As PCR is an exponential process, it can be described by the equation, N_n _= N_0_(1 +ε)^n^, where N_n _is the number of target molecules at cycle n, N_0 _is the initial number of target molecules, ε is the efficiency of amplification and n is the number of cycles. Target amplification efficiency of an assay was determined from the slope of a plot of C_t _(Threshold cycle) versus -log_10 _concentration of the initial number of target molecules. High efficiency of amplification has a slope approaching the value of 3.32 cycles (log_2_10) for every 10-fold dilution of the target. Absolute gene copy numbers for each gene were interpolated from standard curves. All Real-time PCR experiments were compliant with the MIQE (Minimum Information for Publication of Quantitative Real-Time PCR Experiments) guidelines (MIQE form is included as additional file 4).

### Data analysis

Data from four biological replicates were averaged for the analysis. The numbers of biological replicates for 4 hours, 8 hours, 28°C, 37°C subgroups were 30, 32, 29, 40. Gene expression stability analysis using two publicly available software tools, geNorm http://medgen.ugent.be/genorm/ and NormFinder http://www.mdl.dk/ were carried out according to authors' instructions.

## Authors' contributions

KZ, LZ, HPT conceived the study. LZ carried out microarray data analysis. QEL carried out primer design and its validation. KZ, RYZ carried out molecular cloning, cultures, sample treatments. HPT pipetted samples and KZ, LZ, RYZ, HPT carried out qPCR analysis. All authors read and approved the final manuscript.

## Supplementary Material

Additional file 1 **supplementary figures S1-S6 and supplementary tables S1-S3**. supplementary figures and tables for the manuscript. Click here for file

Additional file 2 **list of overexpressed genes in the selected microarray studies**. the file contains the names of overexpressed genes in the selected microarray studies and their functional categories. Click here for file

Additional file 3 **geometric mean calculation table**. an excel table to facilitate calculation of geometric mean of multiple reference genes. Click here for file

Additional file 4 **MIQE checklist**. the checklist to ensure that the study was done according to the MIQE guidelines. Click here for file
