# Supplementary material for: Novel reference genes for quantifying transcriptional responses of Escherichia coli to protein overexpression by quantitative PCR
Source: BMC Mol Biol. 2011 Apr 23;12:18. doi: 10.1186/1471-2199-12-18 (PMC3110127; doi:10.1186/1471-2199-12-18)
Supplement: Additional file 1 — supplementary figures S1-S6 and supplementary tables S1-S3. supplementary figures and tables for the manuscript. [file 1471-2199-12-18-S1.PPTX]

## Slide 1
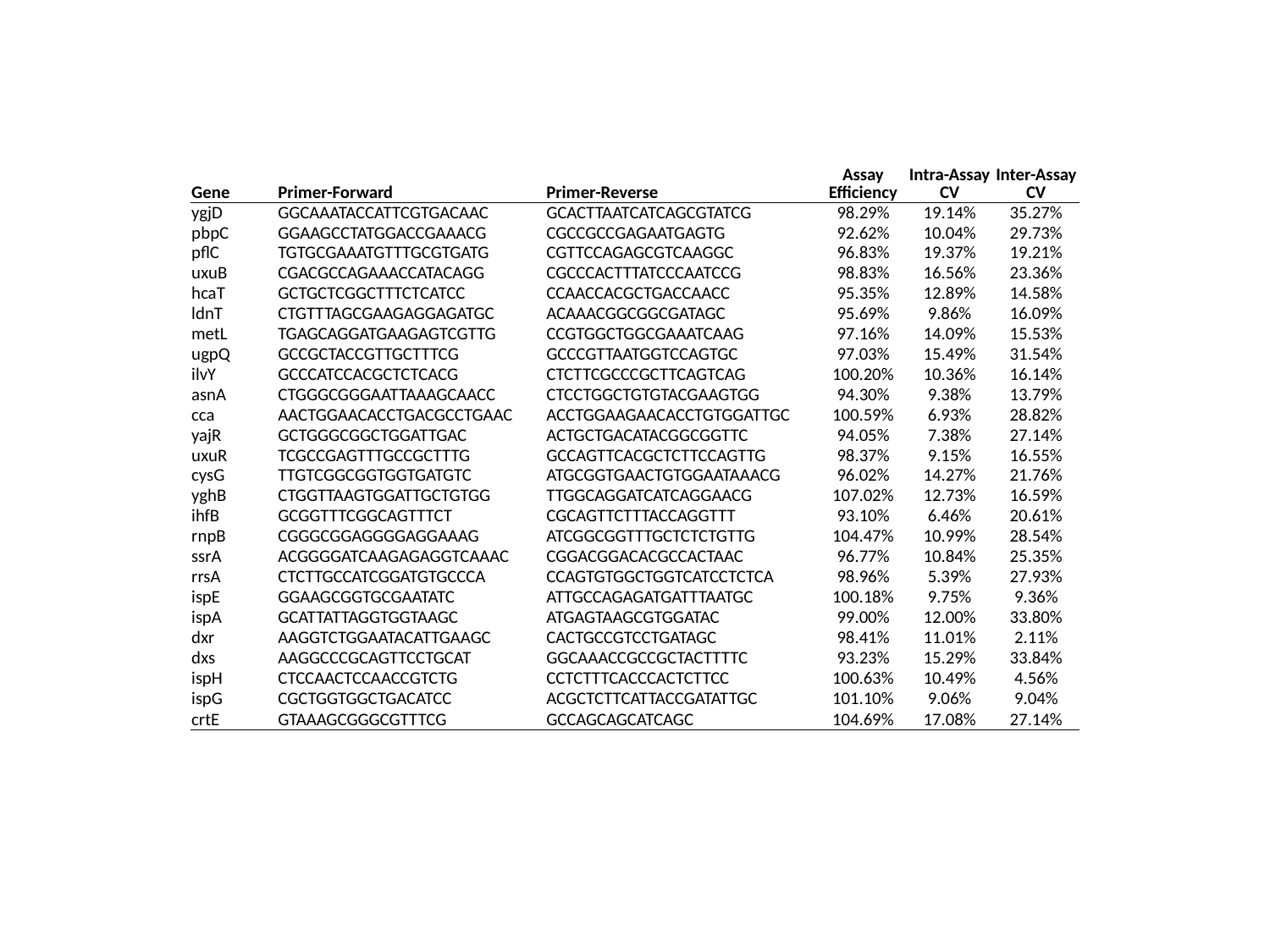

| Gene | Primer-Forward | Primer-Reverse | Assay Efficiency | Intra-Assay CV | Inter-Assay CV |
| --- | --- | --- | --- | --- | --- |
| ygjD | GGCAAATACCATTCGTGACAAC | GCACTTAATCATCAGCGTATCG | 98.29% | 19.14% | 35.27% |
| pbpC | GGAAGCCTATGGACCGAAACG | CGCCGCCGAGAATGAGTG | 92.62% | 10.04% | 29.73% |
| pflC | TGTGCGAAATGTTTGCGTGATG | CGTTCCAGAGCGTCAAGGC | 96.83% | 19.37% | 19.21% |
| uxuB | CGACGCCAGAAACCATACAGG | CGCCCACTTTATCCCAATCCG | 98.83% | 16.56% | 23.36% |
| hcaT | GCTGCTCGGCTTTCTCATCC | CCAACCACGCTGACCAACC | 95.35% | 12.89% | 14.58% |
| ldnT | CTGTTTAGCGAAGAGGAGATGC | ACAAACGGCGGCGATAGC | 95.69% | 9.86% | 16.09% |
| metL | TGAGCAGGATGAAGAGTCGTTG | CCGTGGCTGGCGAAATCAAG | 97.16% | 14.09% | 15.53% |
| ugpQ | GCCGCTACCGTTGCTTTCG | GCCCGTTAATGGTCCAGTGC | 97.03% | 15.49% | 31.54% |
| ilvY | GCCCATCCACGCTCTCACG | CTCTTCGCCCGCTTCAGTCAG | 100.20% | 10.36% | 16.14% |
| asnA | CTGGGCGGGAATTAAAGCAACC | CTCCTGGCTGTGTACGAAGTGG | 94.30% | 9.38% | 13.79% |
| cca | AACTGGAACACCTGACGCCTGAAC | ACCTGGAAGAACACCTGTGGATTGC | 100.59% | 6.93% | 28.82% |
| yajR | GCTGGGCGGCTGGATTGAC | ACTGCTGACATACGGCGGTTC | 94.05% | 7.38% | 27.14% |
| uxuR | TCGCCGAGTTTGCCGCTTTG | GCCAGTTCACGCTCTTCCAGTTG | 98.37% | 9.15% | 16.55% |
| cysG | TTGTCGGCGGTGGTGATGTC | ATGCGGTGAACTGTGGAATAAACG | 96.02% | 14.27% | 21.76% |
| yghB | CTGGTTAAGTGGATTGCTGTGG | TTGGCAGGATCATCAGGAACG | 107.02% | 12.73% | 16.59% |
| ihfB | GCGGTTTCGGCAGTTTCT | CGCAGTTCTTTACCAGGTTT | 93.10% | 6.46% | 20.61% |
| rnpB | CGGGCGGAGGGGAGGAAAG | ATCGGCGGTTTGCTCTCTGTTG | 104.47% | 10.99% | 28.54% |
| ssrA | ACGGGGATCAAGAGAGGTCAAAC | CGGACGGACACGCCACTAAC | 96.77% | 10.84% | 25.35% |
| rrsA | CTCTTGCCATCGGATGTGCCCA | CCAGTGTGGCTGGTCATCCTCTCA | 98.96% | 5.39% | 27.93% |
| ispE | GGAAGCGGTGCGAATATC | ATTGCCAGAGATGATTTAATGC | 100.18% | 9.75% | 9.36% |
| ispA | GCATTATTAGGTGGTAAGC | ATGAGTAAGCGTGGATAC | 99.00% | 12.00% | 33.80% |
| dxr | AAGGTCTGGAATACATTGAAGC | CACTGCCGTCCTGATAGC | 98.41% | 11.01% | 2.11% |
| dxs | AAGGCCCGCAGTTCCTGCAT | GGCAAACCGCCGCTACTTTTC | 93.23% | 15.29% | 33.84% |
| ispH | CTCCAACTCCAACCGTCTG | CCTCTTTCACCCACTCTTCC | 100.63% | 10.49% | 4.56% |
| ispG | CGCTGGTGGCTGACATCC | ACGCTCTTCATTACCGATATTGC | 101.10% | 9.06% | 9.04% |
| crtE | GTAAAGCGGGCGTTTCG | GCCAGCAGCATCAGC | 104.69% | 17.08% | 27.14% |

## Slide 2
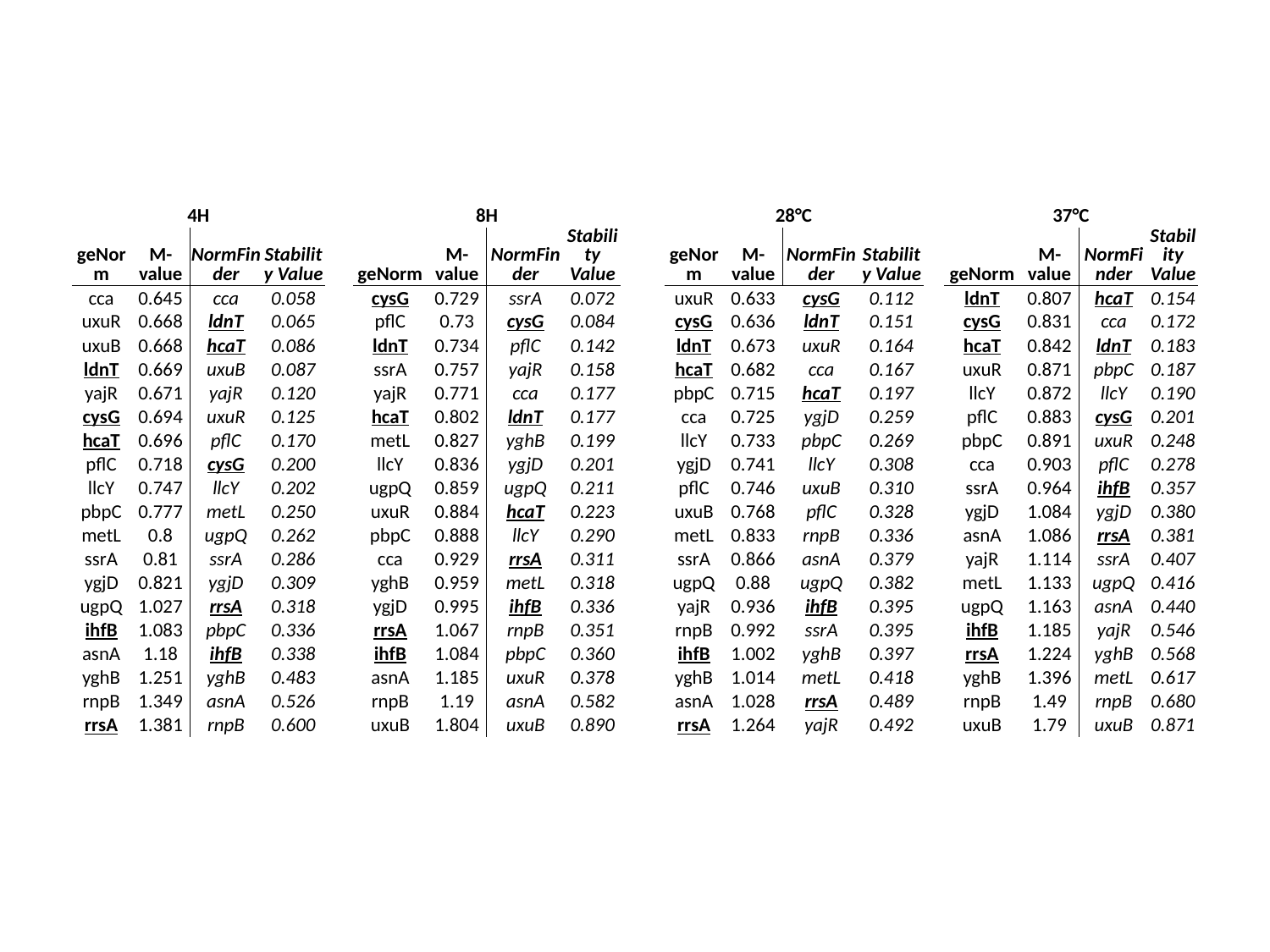

| 4H | | | | | 8H | | | | | 28°C | | | | | 37°C | | | |
| --- | --- | --- | --- | --- | --- | --- | --- | --- | --- | --- | --- | --- | --- | --- | --- | --- | --- | --- |
| geNorm | M-value | NormFinder | Stability Value | | geNorm | M-value | NormFinder | Stability Value | | geNorm | M-value | NormFinder | Stability Value | | geNorm | M-value | NormFinder | Stability Value |
| cca | 0.645 | cca | 0.058 | | cysG | 0.729 | ssrA | 0.072 | | uxuR | 0.633 | cysG | 0.112 | | ldnT | 0.807 | hcaT | 0.154 |
| uxuR | 0.668 | ldnT | 0.065 | | pflC | 0.73 | cysG | 0.084 | | cysG | 0.636 | ldnT | 0.151 | | cysG | 0.831 | cca | 0.172 |
| uxuB | 0.668 | hcaT | 0.086 | | ldnT | 0.734 | pflC | 0.142 | | ldnT | 0.673 | uxuR | 0.164 | | hcaT | 0.842 | ldnT | 0.183 |
| ldnT | 0.669 | uxuB | 0.087 | | ssrA | 0.757 | yajR | 0.158 | | hcaT | 0.682 | cca | 0.167 | | uxuR | 0.871 | pbpC | 0.187 |
| yajR | 0.671 | yajR | 0.120 | | yajR | 0.771 | cca | 0.177 | | pbpC | 0.715 | hcaT | 0.197 | | llcY | 0.872 | llcY | 0.190 |
| cysG | 0.694 | uxuR | 0.125 | | hcaT | 0.802 | ldnT | 0.177 | | cca | 0.725 | ygjD | 0.259 | | pflC | 0.883 | cysG | 0.201 |
| hcaT | 0.696 | pflC | 0.170 | | metL | 0.827 | yghB | 0.199 | | llcY | 0.733 | pbpC | 0.269 | | pbpC | 0.891 | uxuR | 0.248 |
| pflC | 0.718 | cysG | 0.200 | | llcY | 0.836 | ygjD | 0.201 | | ygjD | 0.741 | llcY | 0.308 | | cca | 0.903 | pflC | 0.278 |
| llcY | 0.747 | llcY | 0.202 | | ugpQ | 0.859 | ugpQ | 0.211 | | pflC | 0.746 | uxuB | 0.310 | | ssrA | 0.964 | ihfB | 0.357 |
| pbpC | 0.777 | metL | 0.250 | | uxuR | 0.884 | hcaT | 0.223 | | uxuB | 0.768 | pflC | 0.328 | | ygjD | 1.084 | ygjD | 0.380 |
| metL | 0.8 | ugpQ | 0.262 | | pbpC | 0.888 | llcY | 0.290 | | metL | 0.833 | rnpB | 0.336 | | asnA | 1.086 | rrsA | 0.381 |
| ssrA | 0.81 | ssrA | 0.286 | | cca | 0.929 | rrsA | 0.311 | | ssrA | 0.866 | asnA | 0.379 | | yajR | 1.114 | ssrA | 0.407 |
| ygjD | 0.821 | ygjD | 0.309 | | yghB | 0.959 | metL | 0.318 | | ugpQ | 0.88 | ugpQ | 0.382 | | metL | 1.133 | ugpQ | 0.416 |
| ugpQ | 1.027 | rrsA | 0.318 | | ygjD | 0.995 | ihfB | 0.336 | | yajR | 0.936 | ihfB | 0.395 | | ugpQ | 1.163 | asnA | 0.440 |
| ihfB | 1.083 | pbpC | 0.336 | | rrsA | 1.067 | rnpB | 0.351 | | rnpB | 0.992 | ssrA | 0.395 | | ihfB | 1.185 | yajR | 0.546 |
| asnA | 1.18 | ihfB | 0.338 | | ihfB | 1.084 | pbpC | 0.360 | | ihfB | 1.002 | yghB | 0.397 | | rrsA | 1.224 | yghB | 0.568 |
| yghB | 1.251 | yghB | 0.483 | | asnA | 1.185 | uxuR | 0.378 | | yghB | 1.014 | metL | 0.418 | | yghB | 1.396 | metL | 0.617 |
| rnpB | 1.349 | asnA | 0.526 | | rnpB | 1.19 | asnA | 0.582 | | asnA | 1.028 | rrsA | 0.489 | | rnpB | 1.49 | rnpB | 0.680 |
| rrsA | 1.381 | rnpB | 0.600 | | uxuB | 1.804 | uxuB | 0.890 | | rrsA | 1.264 | yajR | 0.492 | | uxuB | 1.79 | uxuB | 0.871 |

## Slide 3
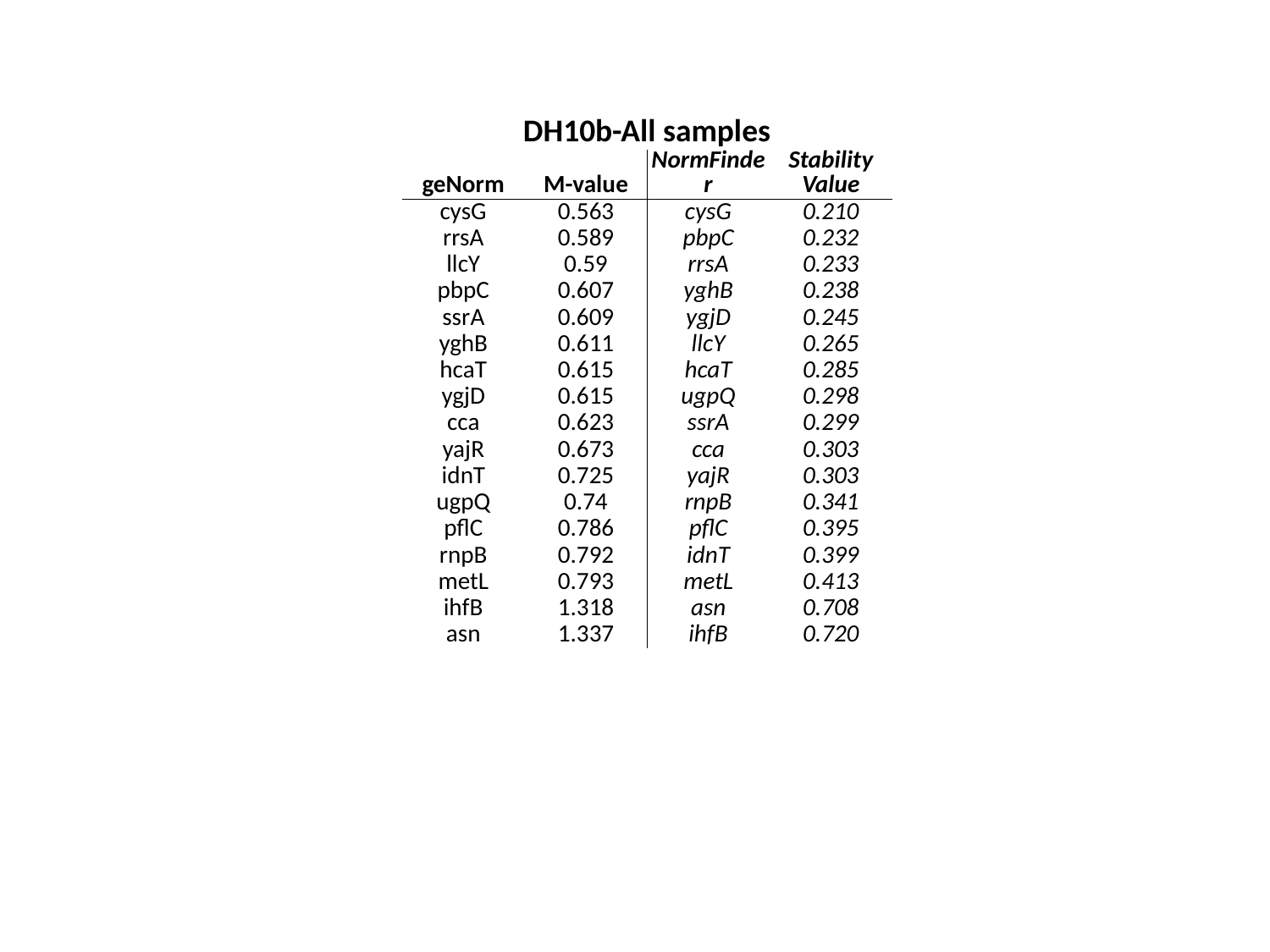

| DH10b-All samples | | | |
| --- | --- | --- | --- |
| geNorm | M-value | NormFinder | Stability Value |
| cysG | 0.563 | cysG | 0.210 |
| rrsA | 0.589 | pbpC | 0.232 |
| llcY | 0.59 | rrsA | 0.233 |
| pbpC | 0.607 | yghB | 0.238 |
| ssrA | 0.609 | ygjD | 0.245 |
| yghB | 0.611 | llcY | 0.265 |
| hcaT | 0.615 | hcaT | 0.285 |
| ygjD | 0.615 | ugpQ | 0.298 |
| cca | 0.623 | ssrA | 0.299 |
| yajR | 0.673 | cca | 0.303 |
| idnT | 0.725 | yajR | 0.303 |
| ugpQ | 0.74 | rnpB | 0.341 |
| pflC | 0.786 | pflC | 0.395 |
| rnpB | 0.792 | idnT | 0.399 |
| metL | 0.793 | metL | 0.413 |
| ihfB | 1.318 | asn | 0.708 |
| asn | 1.337 | ihfB | 0.720 |

## Slide 4
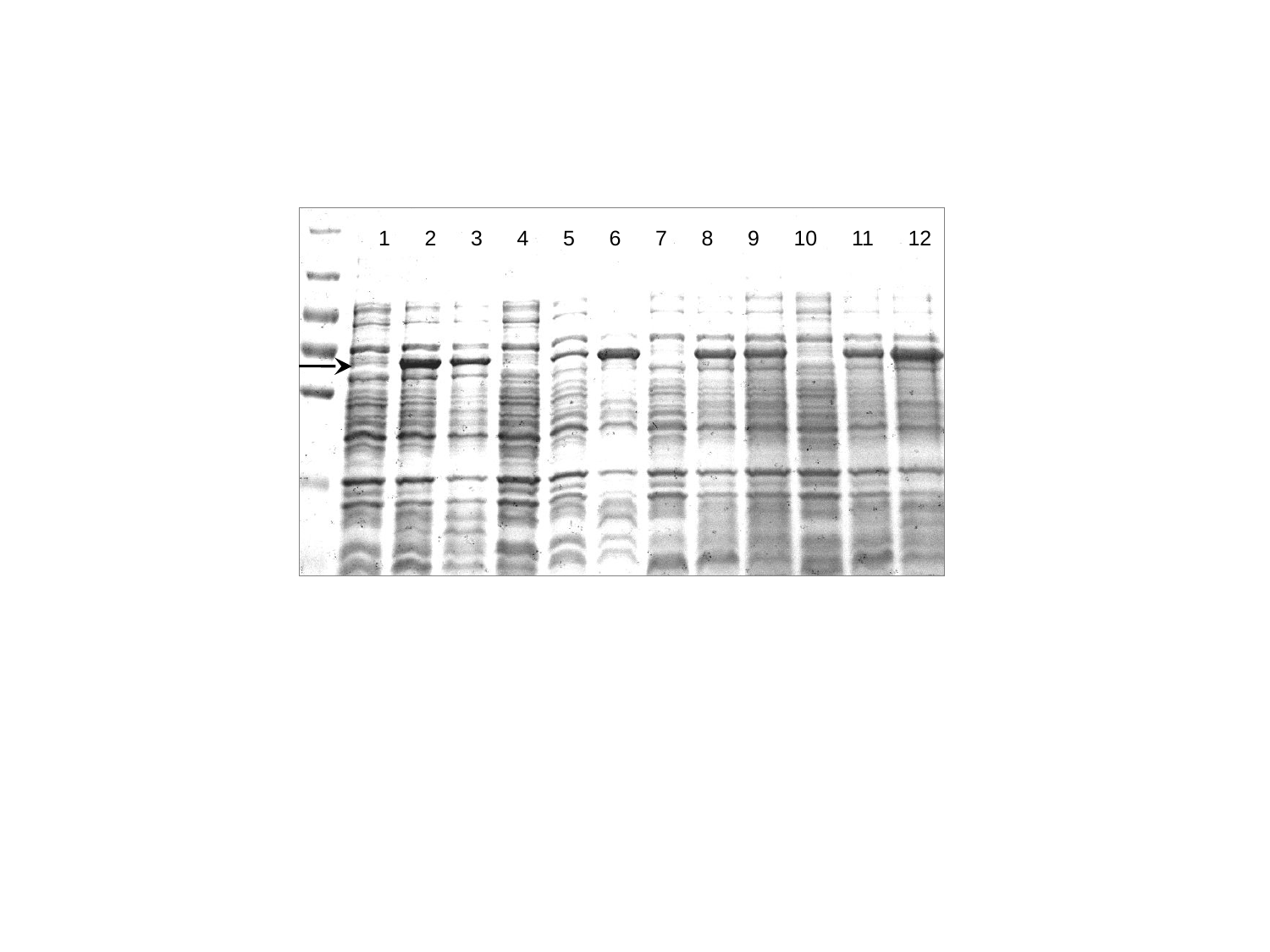

1
2
3
4
5
6
7
8
9
10
11
12

## Slide 5
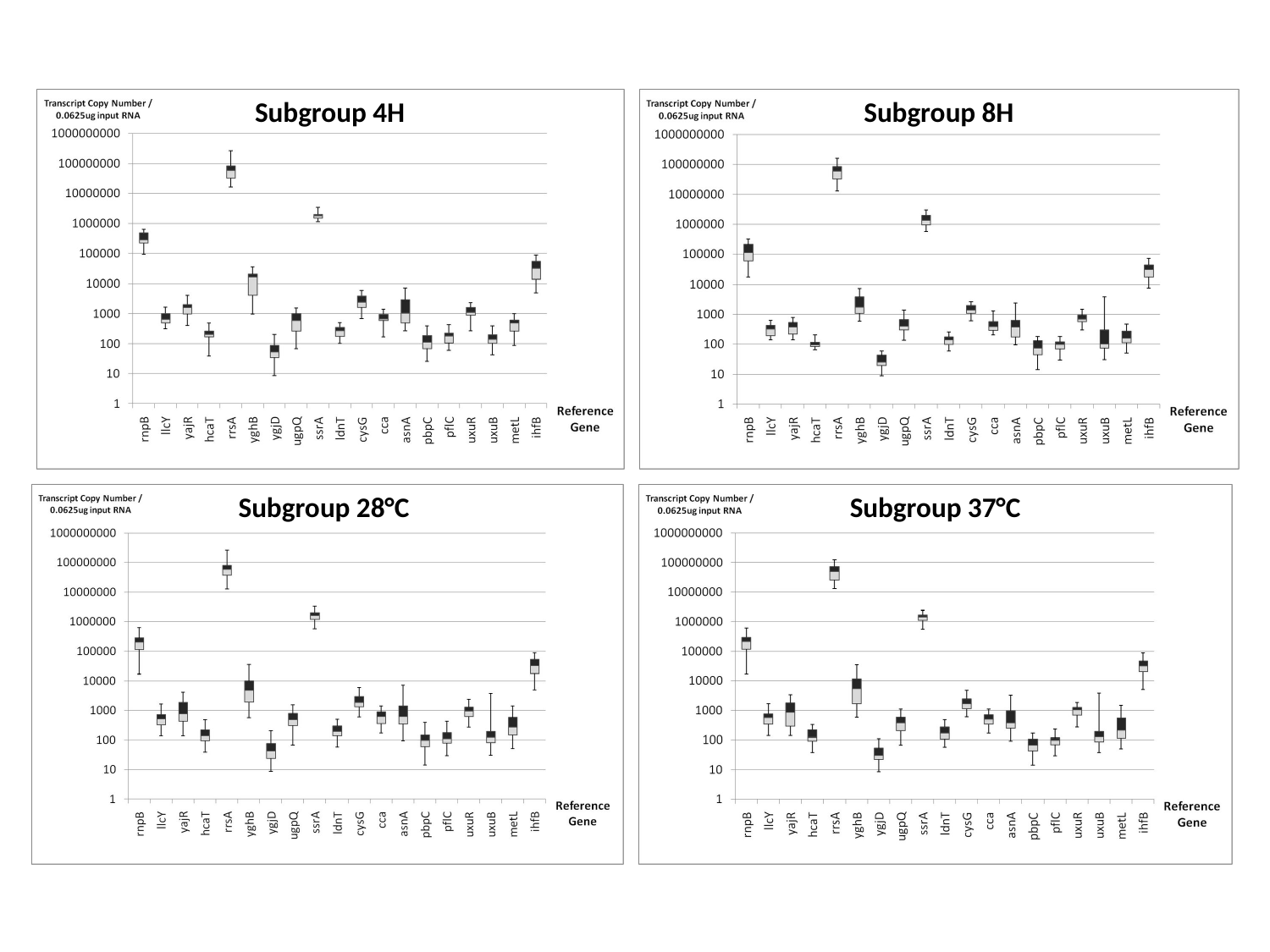

Subgroup 4H
Subgroup 8H
Subgroup 28°C
Subgroup 37°C

## Slide 6
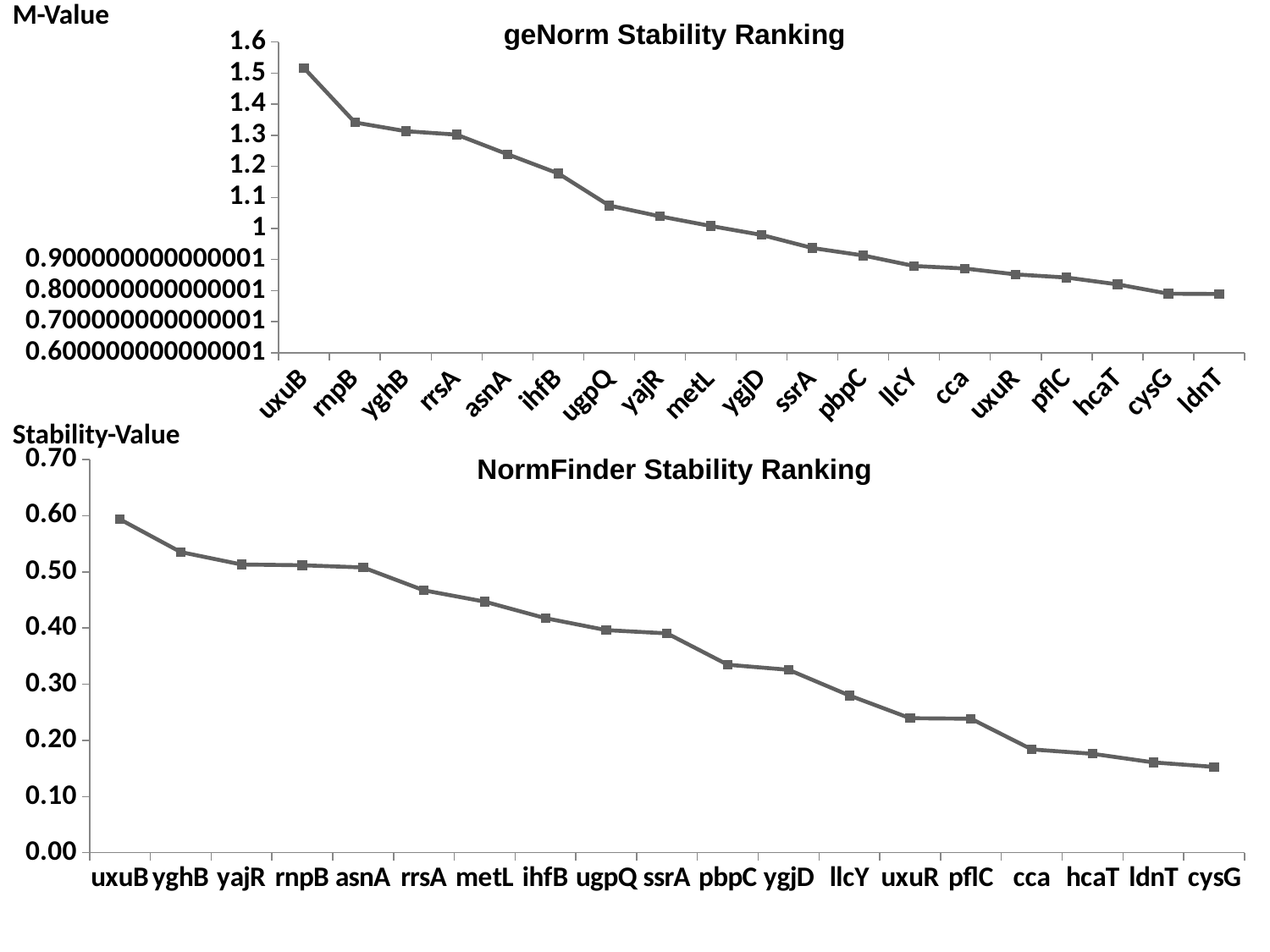

M-Value
geNorm Stability Ranking
### Chart
| Category | |
|---|---|
| uxuB | 1.516 |
| rnpB | 1.341 |
| yghB | 1.312999999999995 |
| rrsA | 1.302 |
| asnA | 1.2389999999999943 |
| ihfB | 1.177 |
| ugpQ | 1.074 |
| yajR | 1.0389999999999946 |
| metL | 1.008 |
| ygjD | 0.9790000000000006 |
| ssrA | 0.9370000000000006 |
| pbpC | 0.913 |
| llcY | 0.8790000000000028 |
| cca | 0.8710000000000024 |
| uxuR | 0.8520000000000006 |
| pflC | 0.8420000000000006 |
| hcaT | 0.8200000000000006 |
| cysG | 0.79 |
| ldnT | 0.789 |Stability-Value
### Chart
| Category | |
|---|---|
| uxuB | 0.5936754654550669 |
| yghB | 0.5353391008778146 |
| yajR | 0.5131746981626943 |
| rnpB | 0.5117678989424476 |
| asnA | 0.5079051359934443 |
| rrsA | 0.4671412113720342 |
| metL | 0.4470449076314093 |
| ihfB | 0.41758896256396244 |
| ugpQ | 0.39635245310953715 |
| ssrA | 0.3905530678239664 |
| pbpC | 0.33467805548619906 |
| ygjD | 0.3256577837570309 |
| llcY | 0.2798642772443692 |
| uxuR | 0.23938761784246324 |
| pflC | 0.2385257708372134 |
| cca | 0.1838837114267969 |
| hcaT | 0.17607584379047841 |
| ldnT | 0.16083266621128037 |
| cysG | 0.15276325243698657 |NormFinder Stability Ranking

## Slide 7
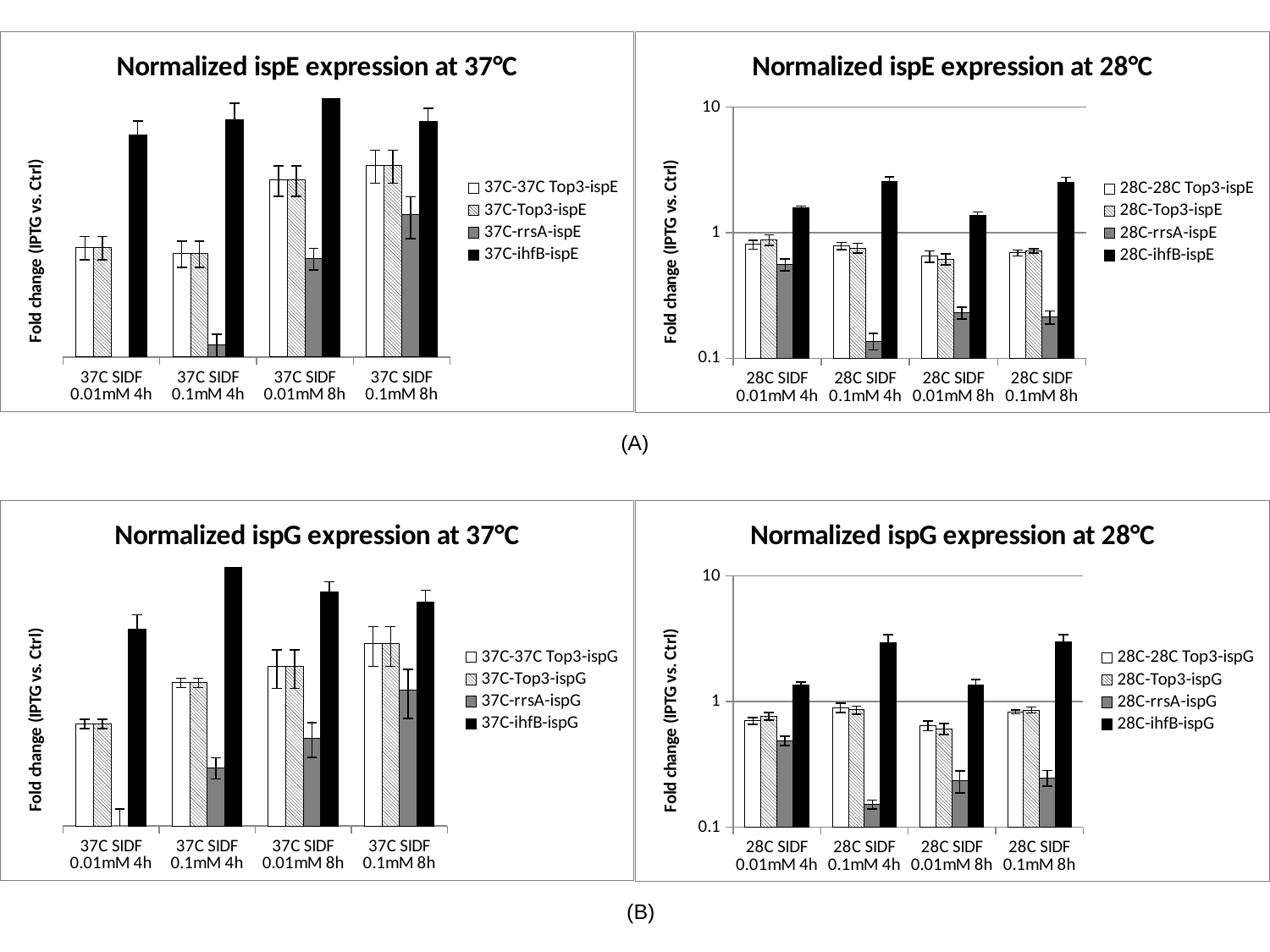

### Chart: Normalized ispE expression at 37°C
| Category | 37C-37C Top3-ispE | 37C-Top3-ispE | 37C-rrsA-ispE | 37C-ihfB-ispE |
|---|---|---|---|---|
| 37C SIDF 0.01mM 4h | 0.7627643096258887 | 0.7627643096258887 | 0.18871764310955624 | 3.181840350799301 |
| 37C SIDF 0.1mM 4h | 0.7072337924430336 | 0.7072337924430336 | 0.22000838779346296 | 3.881527328655014 |
| 37C SIDF 0.01mM 8h | 1.8012390332747397 | 1.8012390332747397 | 0.6612841113314006 | 5.078864587800172 |
| 37C SIDF 0.1mM 8h | 2.172594725599309 | 2.172594725599309 | 1.151146473757006 | 3.7960238949637635 |
### Chart: Normalized ispE expression at 28°C
| Category | 28C-28C Top3-ispE | 28C-Top3-ispE | 28C-rrsA-ispE | 28C-ihfB-ispE |
|---|---|---|---|---|
| 28C SIDF 0.01mM 4h | 0.8050067923574157 | 0.8764437102646068 | 0.5567705312419549 | 1.566850700153352 |
| 28C SIDF 0.1mM 4h | 0.7840540592462145 | 0.7533404012147834 | 0.13692514569233696 | 2.549256426595374 |
| 28C SIDF 0.01mM 8h | 0.6470335396888399 | 0.6163058312545178 | 0.23070428341918342 | 1.380726544491982 |
| 28C SIDF 0.1mM 8h | 0.6891826768356767 | 0.7154545792568572 | 0.2119573051118898 | 2.5023990960946554 |(A)
### Chart: Normalized ispG expression at 37°C
| Category | 37C-37C Top3-ispG | 37C-Top3-ispG | 37C-rrsA-ispG | 37C-ihfB-ispG |
|---|---|---|---|---|
| 37C SIDF 0.01mM 4h | 0.7150806796196196 | 0.7150806796196196 | 0.1706601573613694 | 2.6952343800357403 |
| 37C SIDF 0.1mM 4h | 1.2696082180624357 | 1.2696082180624357 | 0.38619988635125124 | 6.382724652602646 |
| 37C SIDF 0.01mM 8h | 1.591267114997346 | 1.591267114997346 | 0.58502511466453 | 4.52020702631587 |
| 37C SIDF 0.1mM 8h | 2.18468694824474 | 2.18468694824474 | 1.1471370776692957 | 3.9062906076446677 |
### Chart: Normalized ispG expression at 28°C
| Category | 28C-28C Top3-ispG | 28C-Top3-ispG | 28C-rrsA-ispG | 28C-ihfB-ispG |
|---|---|---|---|---|
| 28C SIDF 0.01mM 4h | 0.7008192970304279 | 0.7663398388751921 | 0.4879275402711735 | 1.3586187064744932 |
| 28C SIDF 0.1mM 4h | 0.8932189810889354 | 0.8562323372073253 | 0.1522361169715359 | 2.943716733849827 |
| 28C SIDF 0.01mM 8h | 0.6433055846599836 | 0.6076638102759915 | 0.234280340655271 | 1.3643786527110637 |
| 28C SIDF 0.1mM 8h | 0.8274093568836347 | 0.85605790335793 | 0.24763447415749035 | 2.997015209195916 |(B)

## Slide 8
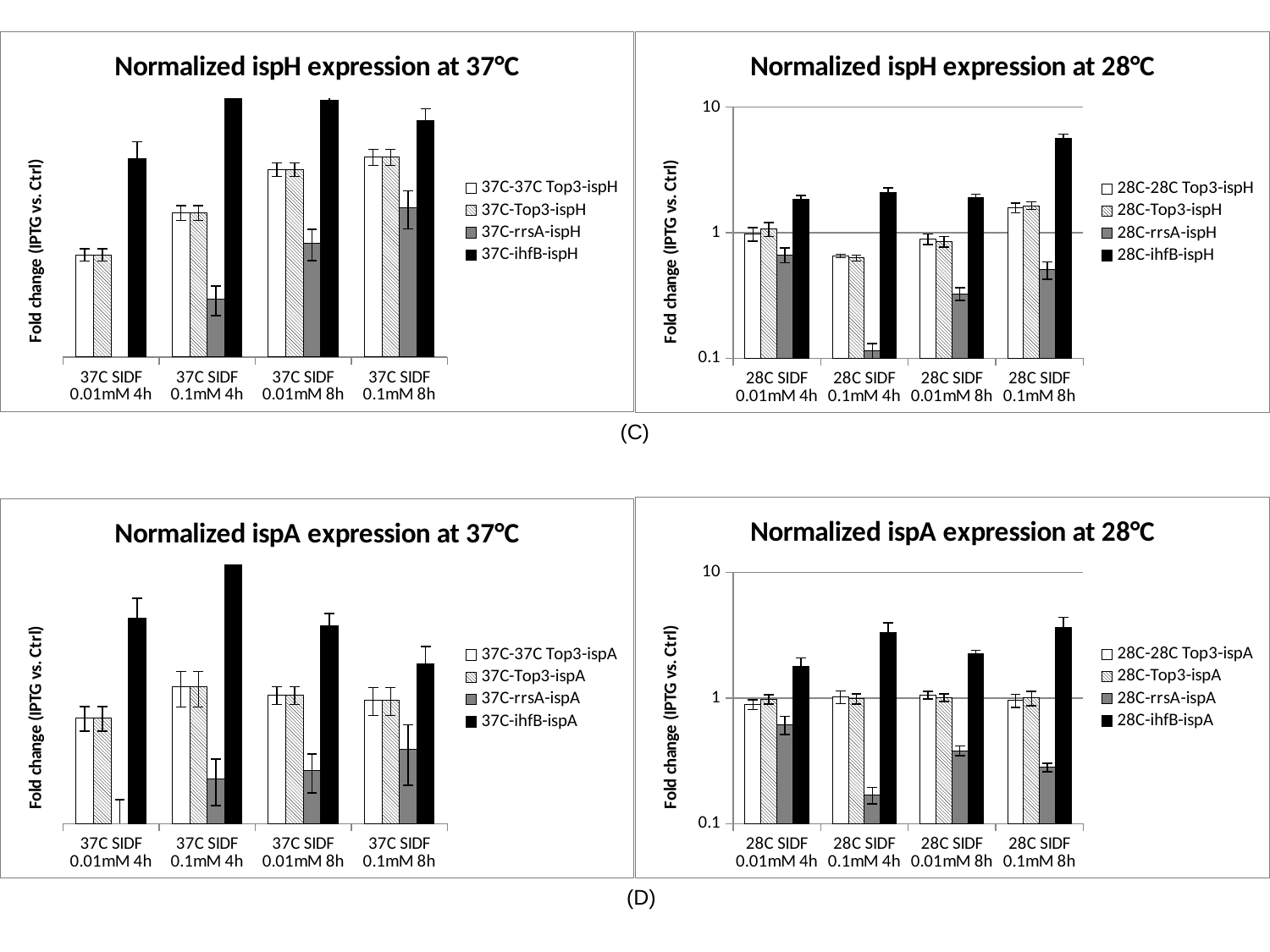

### Chart: Normalized ispH expression at 37°C
| Category | 37C-37C Top3-ispH | 37C-Top3-ispH | 37C-rrsA-ispH | 37C-ihfB-ispH |
|---|---|---|---|---|
| 37C SIDF 0.01mM 4h | 0.6076130196177539 | 0.6076130196177539 | 0.14782969179949093 | 2.3014354505754677 |
| 37C SIDF 0.1mM 4h | 1.0893039642226647 | 1.0893039642226647 | 0.3286673155331523 | 5.2849768682118645 |
| 37C SIDF 0.01mM 8h | 1.9806778589116865 | 1.980677858911687 | 0.7120325682875055 | 5.162657639083782 |
| 37C SIDF 0.1mM 8h | 2.3464492200711438 | 2.3464492200711438 | 1.1707983880251376 | 3.8632699405360795 |
### Chart: Normalized ispH expression at 28°C
| Category | 28C-28C Top3-ispH | 28C-Top3-ispH | 28C-rrsA-ispH | 28C-ihfB-ispH |
|---|---|---|---|---|
| 28C SIDF 0.01mM 4h | 0.9763404470838508 | 1.0664375397204073 | 0.6648145406432067 | 1.8396921062818141 |
| 28C SIDF 0.1mM 4h | 0.6508908702713624 | 0.6287315653743949 | 0.11383147652132601 | 2.0950003197737033 |
| 28C SIDF 0.01mM 8h | 0.88735746031657 | 0.8474262078955753 | 0.3265512448347293 | 1.906113889027869 |
| 28C SIDF 0.1mM 8h | 1.5774743439446606 | 1.6390504008452158 | 0.5051761390174955 | 5.604219557740195 |(C)
### Chart: Normalized ispA expression at 28°C
| Category | 28C-28C Top3-ispA | 28C-Top3-ispA | 28C-rrsA-ispA | 28C-ihfB-ispA |
|---|---|---|---|---|
| 28C SIDF 0.01mM 4h | 0.8867753367921462 | 0.9802335420005771 | 0.6135846507982866 | 1.7786341615943115 |
| 28C SIDF 0.1mM 4h | 1.0188735870209658 | 0.9828052773989486 | 0.1683324631013685 | 3.3405499226530737 |
| 28C SIDF 0.01mM 8h | 1.0575276184966378 | 1.006650616953848 | 0.3813795025912135 | 2.2705021449668155 |
| 28C SIDF 0.1mM 8h | 0.9537132005197659 | 0.9988026294937267 | 0.28111660024673435 | 3.647136288206213 |
### Chart: Normalized ispA expression at 37°C
| Category | 37C-37C Top3-ispA | 37C-Top3-ispA | 37C-rrsA-ispA | 37C-ihfB-ispA |
|---|---|---|---|---|
| 37C SIDF 0.01mM 4h | 1.2136068705324345 | 1.2136068705324345 | 0.28927945638308794 | 4.654190032975886 |
| 37C SIDF 0.1mM 4h | 1.8403828413352759 | 1.8403828413352754 | 0.5313710505573238 | 9.613581236996675 |
| 37C SIDF 0.01mM 8h | 1.6530516484928677 | 1.653051648492868 | 0.5919028840796912 | 4.2004708065053435 |
| 37C SIDF 0.1mM 8h | 1.540155513112668 | 1.540155513112668 | 0.7947309629282779 | 2.5075795848273157 |(D)

## Slide 9
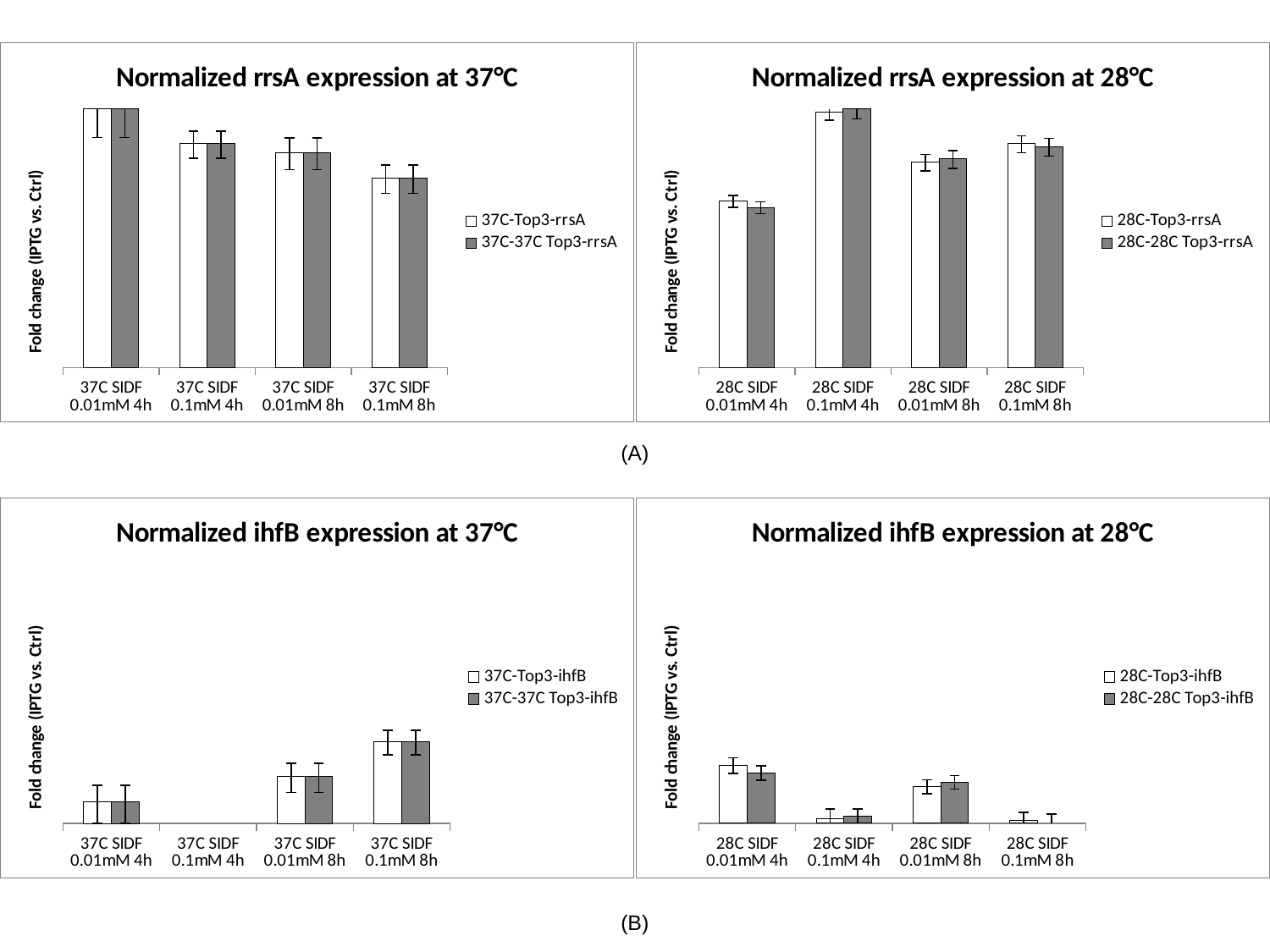

### Chart: Normalized rrsA expression at 37°C
| Category | 37C-Top3-rrsA | 37C-37C Top3-rrsA |
|---|---|---|
| 37C SIDF 0.01mM 4h | 4.77990158944244 | 4.77990158944244 |
| 37C SIDF 0.1mM 4h | 3.1317085479302182 | 3.1317085479302182 |
| 37C SIDF 0.01mM 8h | 2.8206743830558367 | 2.8206743830558367 |
| 37C SIDF 0.1mM 8h | 2.0708281769044627 | 2.0708281769044627 |
### Chart: Normalized rrsA expression at 28°C
| Category | 28C-Top3-rrsA | 28C-28C Top3-rrsA |
|---|---|---|
| 28C SIDF 0.01mM 4h | 1.5354346079721575 | 1.398327722910305 |
| 28C SIDF 0.1mM 4h | 5.542998397495945 | 5.782188953522287 |
| 28C SIDF 0.01mM 8h | 2.6857513063111456 | 2.8159048855240387 |
| 28C SIDF 0.1mM 8h | 3.506382859277288 | 3.357444335706888 |(A)
### Chart: Normalized ihfB expression at 37°C
| Category | 37C-Top3-ihfB | 37C-37C Top3-ihfB |
|---|---|---|
| 37C SIDF 0.01mM 4h | 0.2651064222850955 | 0.2651064222850955 |
| 37C SIDF 0.1mM 4h | 0.20002234794796467 | 0.20002234794796467 |
| 37C SIDF 0.01mM 8h | 0.36894795225536847 | 0.36894795225536847 |
| 37C SIDF 0.1mM 8h | 0.5809423775614391 | 0.580942377561439 |
### Chart: Normalized ihfB expression at 28°C
| Category | 28C-Top3-ihfB | 28C-28C Top3-ihfB |
|---|---|---|
| 28C SIDF 0.01mM 4h | 0.5658176129540476 | 0.5189704729237178 |
| 28C SIDF 0.1mM 4h | 0.306752448582273 | 0.3156667737504031 |
| 28C SIDF 0.01mM 8h | 0.4437453798386533 | 0.46645275963496874 |
| 28C SIDF 0.1mM 8h | 0.3001237233163572 | 0.2905730169016023 |(B)

## Slide 10
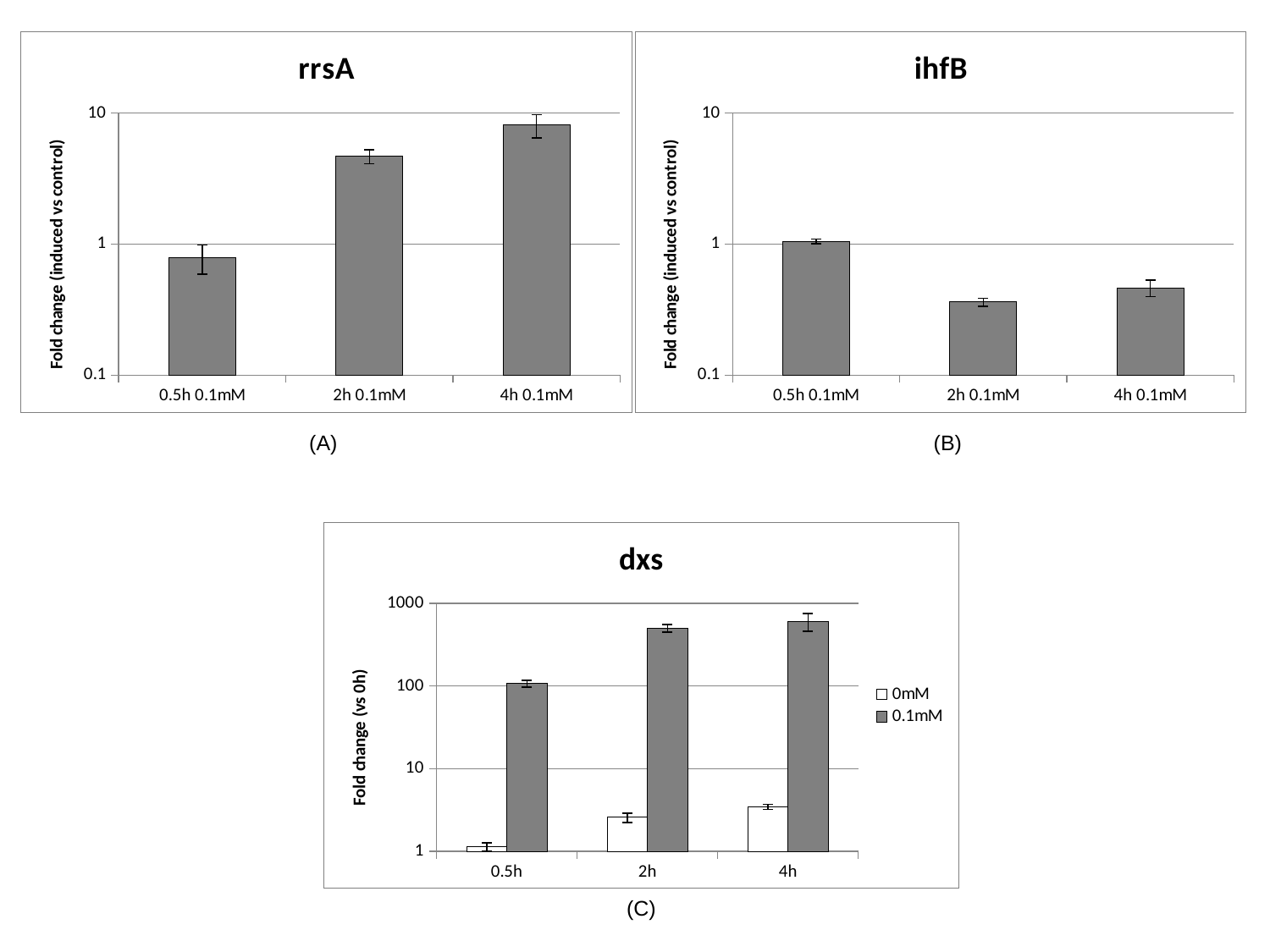

### Chart:
| Category | rrsA |
|---|---|
| 0.5h 0.1mM | 0.7863668992882206 |
| 2h 0.1mM | 4.670252471691796 |
| 4h 0.1mM | 8.097517112728003 |
### Chart:
| Category | ihfB |
|---|---|
| 0.5h 0.1mM | 1.052335415810706 |
| 2h 0.1mM | 0.3608939154003933 |
| 4h 0.1mM | 0.46436491892764986 |(A)
(B)
### Chart: dxs
| Category | 0mM | 0.1mM |
|---|---|---|
| 0.5h | 1.1481310711875354 | 106.9200575590178 |
| 2h | 2.576201161061977 | 501.4667353040346 |
| 4h | 3.4603709206578412 | 604.7549641942355 |(C)
